# Exploring vascular access survival in prevalent thrice-weekly in-centre nocturnal haemodialysis patients

**DOI:** 10.1007/s40620-025-02431-1

**Published:** 2025-09-26

**Authors:** Katherine L. Hull, Ann Bugeja, Matthew P. M. Graham-Brown, Lindsay Reid, Aiden J. Smith, Brigit C. van Jaarsveld, James O. Burton

**Affiliations:** 1 Department of Cardiovascular Sciences, University of Leicester, Leicester, UK; 2 John Walls Renal Unit, University Hospitals of Leicester NHS Trust, Leicester, UK; 3 The Ottawa Hospital, Ottawa Hospital Research Institute, and Kidney Research Centre, Ottawa, ON Canada; 4 University of Ottawa, Ottawa, ON Canada; 5 School of Sport, Exercise and Health Sciences, Loughborough University, Loughborough, UK; 6 Leicester Kidney Lifestyle Team, Department of Population Health Sciences, University of Leicester, Leicester, UK; 7 Department of Population Health Sciences, University of Leicester, Leicester, UK; 8 Amsterdam University Medical Centre, Nephrology, and Research Institute Amsterdam Cardiovascular Sciences, Amsterdam, The Netherlands; 9 Leicester British Heart Foundation Centre of Research Excellence, Leicester, UK

**Keywords:** Kidney failure, In-centre nocturnal haemodialysis, Retrospective, Vascular access

## Abstract

**Background:**

This study explores vascular access complications in patients established on in-centre nocturnal haemodialysis (INHD) compared to conventional haemodialysis.

**Methods:**

This was a retrospective cohort study; patients acted as their own control. Data were collected from three centres. Adults established on INHD (intervention) preceded by usual daytime haemodialysis (control) were eligible. Data were collected between 01/01/2009 and 12/31/2021. The data collection period was up to 12 months for both control and intervention periods.

The primary outcome was a composite of outcomes related to vascular access complications: hospitalisation, intervention, change in vascular access modality, change in dialysis modality and death. The primary outcome was evaluated by time-to-event rate in days using Kaplan–Meier plots. Statistical significance was accepted at a *P* < 0.05*.*

**Results:**

One hundred forty-five individuals were included: median age was 52.0 years (IQR 36.0–65.0), 71.0% (*n* = 103) were male, and 57.2% (*n* = 83) were White.

The primary outcome occurred in 24.1% (*n* = 35) during the intervention and in 25.5% (*n* = 37) during the control period (*P* = 0.875). The 12-month vascular access survival probability was 73.4% (95%CI 65.8–81.0%) for the intervention and 70.6% (95%CI 62.4%-78.8%) for the control period.

During the intervention period, arteriovenous grafts were associated with lower vascular access survival (*P* < 0.001). Regular vitamin K antagonist was associated with a lower 12-month vascular access survival for both the intervention (*P* = 0.044) and the control periods (*P* < 0.001).

**Conclusion:**

There does not appear to be an increased risk to vascular access events for INHD compared to daytime haemodialysis. Vascular access type and regular anticoagulation were associated with a reduced vascular access survival probability.

**Graphical abstract:**

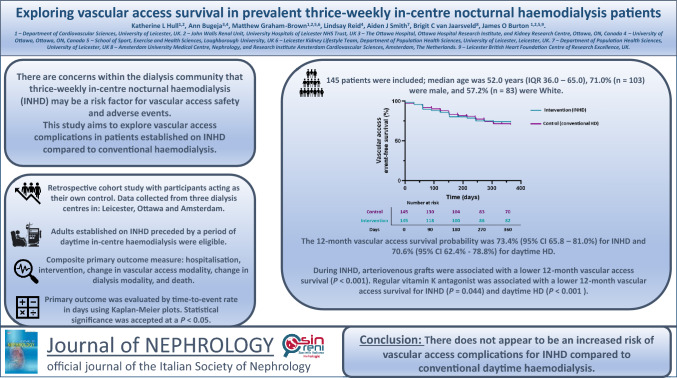

**Supplementary Information:**

The online version contains supplementary material available at 10.1007/s40620-025-02431-1.

## Introduction

There are concerns within the dialysis community regarding the vascular access safety of in-centre nocturnal haemodialysis (INHD) and this may be impacting its implementation. It is estimated that only 5% of dialysis centres offer INHD [1]. The UK Kidney Association haemodialysis guidelines warn augmented haemodialysis schedules may cause potential harm, including an impact on vascular access complications and vascular access survival [2].

In the Frequent Haemodialysis Network (FHN) trial, there was a trend towards increased vascular access events with nocturnal home haemodialysis (six times a week, ≥ 6 h per session) [3]. Meta-analysis of the FHN Nocturnal trial with the ACTIVE dialysis trial (home or in-centre haemodialysis, ≥ 3 sessions a week, total weekly dialysis hours ≥ 24) and the Alberta trial (five to six sessions a week of nocturnal home haemodialysis, ≥ 6 h per session), did not demonstrate a significant difference in the relative risk of vascular access adverse events (RR = 1.25, 95% CI 0.88–1.77, *P* = 0.21) [4]. However, in Cornelis et al.’s review incorporating the randomised controlled trial (RCT) data of the FHN trials and Alberta trial with prospective and retrospective cohort studies, the pooled findings demonstrated *intensive* (≥ 3 times a week) haemodialysis was associated with a higher vascular access event rate than conventional haemodialysis (difference = 6.7 events per 100 patient-years, *P* = 0.009) [5].

The impact of nocturnal dialysis regimens on vascular access are inconclusive but have generated concern nonetheless. Most studies do not include *thrice*-weekly, extended-hours INHD as the intervention. We hypothesise there is no significant difference in vascular access adverse events between thrice-weekly extended-hours INHD and thrice-weekly in-centre daytime haemodialysis.

## Methods

### Study design

This study was a retrospective cohort study with participants acting as their own control. Data were collected from three dialysis centres: Leicester Renal Network (registered project number: 12494), UK, offering INHD at one hospital-based and two satellite dialysis units; The Ottawa Hospital (project number: 20230336-01H), Canada, offering INHD in a hospital-based dialysis unit; and Nephrocare DiaPriva Dialysis Centre (project number: DP20240516), Netherlands, offering INHD at a satellite dialysis unit.

### Participants

Eligible participants fulfilled the following inclusion criteria:

-  ≥ 18 years of age;
- Maintenance haemodialysis, defined as ≥ 3 months of haemodialysis;
- Established on INHD, defined as ≥ 30 consecutive days of INHD, between January 1st, 2009 to December 31st, 2021;
- Established vascular access i.e., tunnelled catheter, arteriovenous fistula (AVF), arteriovenous graft (AVG). Patients receiving haemodialysis via a non-tunnelled central line were not eligible.

### Data collection

Time Point Zero (T0) was defined as the commencement of INHD. Demographic data were collected at T0. The intervention period for data collection was T1: up to 12 months of INHD. The control period to data collection was T-1: up to 12 months prior to commencing INHD during which the participant received conventional in-centre daytime haemodialysis. Vascular access type was collected at the beginning of the control period (T-1) and commencement of INHD (T0). The control and intervention periods were consecutive and all participants received conventional haemodialysis before INHD.

An overview of the study design is available in Supplementary Fig. 1.

### Comparators

The intervention was extended-hours INHD, defined as six to eight hours of haemodialysis at a dialysis unit thrice-weekly and overnight.

The control was conventional daytime in-centre haemodialysis, defined as three and a half to five hours of haemodialysis at a dialysis unit thrice-weekly during the day.

### Primary and secondary outcomes

The primary outcome was a composite index of:

- Hospitalisation due to vascular access complication;
- Radiological or surgical intervention to manage vascular access complication;
- Switch in vascular access modality due to vascular access complication;
- Switch in dialysis modality (i.e., change to peritoneal dialysis) due to vascular access complication;
- Death due to vascular access complication.

The secondary outcomes were:

- Incidence rates of: needle dislodgement, post-dialysis AVF/AVG bleed, spontaneous AVF/AVG bleed;
- Proportion of patients experiencing: thrombosis, stenosis, aneurysm, infection;
- Proportion of patients requiring: medical thrombolysis, radiological intervention, surgical intervention;
- Incidence rate of hospitalisation due to any vascular access complication.

The primary and secondary outcome data were collected from a retrospective review of medical and dialysis unit records at the participating centres. This meant the definition of the outcomes were not predefined for the study but were determined by the actual events and diagnoses recorded in medical and dialysis units notes at the participating centres.

### Vascular access monitoring at participating centres

Each participating centre carries out vascular access monitoring as part of routine dialysis care: there is no additional or alternative vascular access monitoring for INHD compared to daytime in-centre haemodialysis.

In the Leicester Renal Network, blood temperature monitoring is completed once a month to evaluate vascular access flow and recirculation. Referral for further assessment (typically with a fistulogram) is indicated by a decrease in AVF/AVG blood flow of 25% or recirculation of ≥ 5%.

At the Ottawa Hospital, transonic ultrasound dilution technology is used once a month to evaluate vascular access flow and recirculation in AVF/AVGs, as well as in a new AVF/AVG and after surgical or radiological intervention. If access flow decreases ≥ 20% from the previous measurement, or if AVG flow is < 600 mL/min or AVF flow is < 500 mL/min, or there is a downward trend in the blood flow rate, the Nephrologist and Dialysis Access Registered Nurse or Advanced Practice Nurses are notified without delay for consideration of further assessment.

In the Nephrocare DiaPriva Dialysis Centre, vascular access flow is also measured with transonic ultrasound technology every month in AVGs and every two to three months in AVFs; the frequency of assessments is increased if vascular access flow is reduced or increased by ≥ 25% or when an AVF/AVG stenosis or thrombosis has occurred previously. Referral for further imaging occurs if vascular access flow is < 500 mL/min or there is a continued decrease of ≥ 25% in vascular access flow due to the likelihood that stenosis is progressing.

### Vascular access venepuncture at participating centres

At each participating centre, the predominant venepuncture technique is rope ladder. There are individual circumstances whereby the button hole technique has been used. Data regarding each participant’s venepuncture technique were not available for collection from the medical records; it has been presumed that the standard approach of rope ladder needling was used.

### Covariates of interest

The covariates of interest include age, dialysis vintage, smoking status, type of vascular access, anticoagulation prescription, antiplatelet prescription, and co-morbidities (diabetes mellitus, ischaemic heart disease, heart failure, stroke and transient ischaemic attack, peripheral vascular disease, hypertension, obesity and active malignancy).

### Statistical analysis

Descriptive statistics are presented as mean (with SD) or median (with IQR) for continuous variables and as frequencies with percentages for categorical variables. Continuous data distributions were evaluated using histogram plots. The primary outcome was evaluated by time-to-event rate in days using Kaplan–Meier plots. Potential relationships between the covariates of interest (as listed above) and the primary composite outcome were explored for the intervention (INHD) and control (conventional haemodialysis) periods by log-rank test. With regard to the continuous covariates of age and dialysis vintage, the data were categorised into two groups according to the median value to complete the log-rank test. The log rank test was chosen to explore the potential relationship between the covariates of interest and the primary outcome because the paired nature of the data meant assumptions of techniques, such as the Cox Proportional Hazards Regression analysis (e.g., hazards are proportional/the relative hazard remains constant over time), could not be met.

For secondary outcomes, incidence rates (person-years) and frequencies with percentages were determined for continuous and categorical variables, respectively. Incidence rates were compared between the intervention and control periods using the Wilcoxon signed-rank test. The frequency of categorical variables between the intervention and control periods was compared using the McNemar test. Statistical significance was accepted at a *P* < 0.05*.*

Statistical analysis of data was performed using the Statistical Package for Social Sciences (SPSS for Windows, Version 28.0, IBM, New York, USA). Kaplan–Meier plots were created using GraphPad Prism (version 9.5.1 for Windows, GraphPad Software, Boston, Massachusetts USA).

### Confidentiality, data handling and storage

Data collection involved a retrospective review of clinical records. All data were treated in confidence, de-identified (pseudo-anonymised), with storage and analysis occurring on a password protected and encrypted laptop. Individual participant consent was not required at the collaborating centres for retrospective data collection from clinical records.

## Results

### Participant characteristics

One hundred forty-five patients were included in this study, of whom 62 were from the UK dialysis centre, 55 were from the Canadian dialysis centre, and 28 from the Dutch dialysis centre. The median age was 52.0 years (IQR 36.0–65.0), 71.0% (*n* = 103) were male, 57.2% (*n* = 83) were White and the median dialysis vintage was 549.0 days (IQR 243.5–1415.0). The full participant characteristics are reported in Table 1.

**Table 1:** Demographics of the study cohort

Variable	Cohort *n* = 145	
*Age* (years)	52.0 (IQR 36.0–65.0)	
*Sex*		
Male	103 (71.0%)	
Female	42 (29.0%)	
*Race*		
White	83 (57.2%)	
Black	18 (12.4%)	
Asian	10 (6.9%)	
Multiple	6 (4.1%)	
Other	2 (1.4%)	
Unknown	26 (17.9%)	
*Haemodialysis vintage* (days)	549.0 (IQR 243.5–1415)	
*Smoking status*		
Current smoker	28 (19.3%)	
Previous smoker	39 (26.9%)	
Never smoked	44 (30.3%)	
Unknown	34 (23.4%)	
*Diabetes mellitus*		
No diagnosis	88 (60.7%)	
Type 1	10 (6.9%)	
Type 2	45 (31.0%)	
Other/unknown type	2 (1.4%)	
*Ischaemic heart disease*		
Yes	34 (23.4%)	
No	111 (76.6%)	
*Heart failure*		
Yes	23 (15.9%)	
No	122 (84.1%)	
*Stroke or Transient ischaemic attack*		
Yes	9 (6.2%)	
No	136 (93.8%)	
*Peripheral vascular disease*		
Yes	13 (9.0%)	
No	132 (91.0%)	
*Hypertension*		
Yes	110 (75.9%)	
No	35 (24.1%)	
*Obesity*		
Yes	50 (34.5%)	
No	95 (65.5%)	
*Active malignancy*		
Yes	11 (7.6%)	
No	134 (92.4%)	
*Antiplatelet*		
No antiplatelet	101 (69.7%)	
Single antiplatelet	40 (27.6%)	
Dual antiplatelet	4 (2.8%)	
*Anticoagulation*		
No anticoagulation	127 (87.6%)	
Vitamin K antagonist	17 (11.7%)	
Low molecular weight heparin	1 (0.7%)	
*Vascular access*	*Control*	*Intervention*
Tunnelled catheter	72 (49.7%)	58 (40.0%)
Arteriovenous fistula	65 (44.8%)	78 (53.8%)
Arteriovenous graft	8 (5.5%)	9 (6.2%)
*Weekly haemodialysis* (hours)	12.0 (IQR 12.0–12.0)	24.0 (IQR 21.0–24.0)
*Length of follow-up* (days)	365.0 (IQR 243.0–365.0)	365.0 (IQR 335.0–365.0)

Data are presented as mean ± standard deviation, median (interquartile range) and percentage (frequency) as appropriate

### Primary outcome

The primary outcome occurred in 24.1% (*n* = 35) of patients during the intervention period and in 25.5% (*n* = 37) of patients during the control period. There was no significant difference in the proportion of patients experiencing the primary outcome (*P* = 0.875). There were no reported deaths due to vascular access adverse events.

**Fig. 1:**
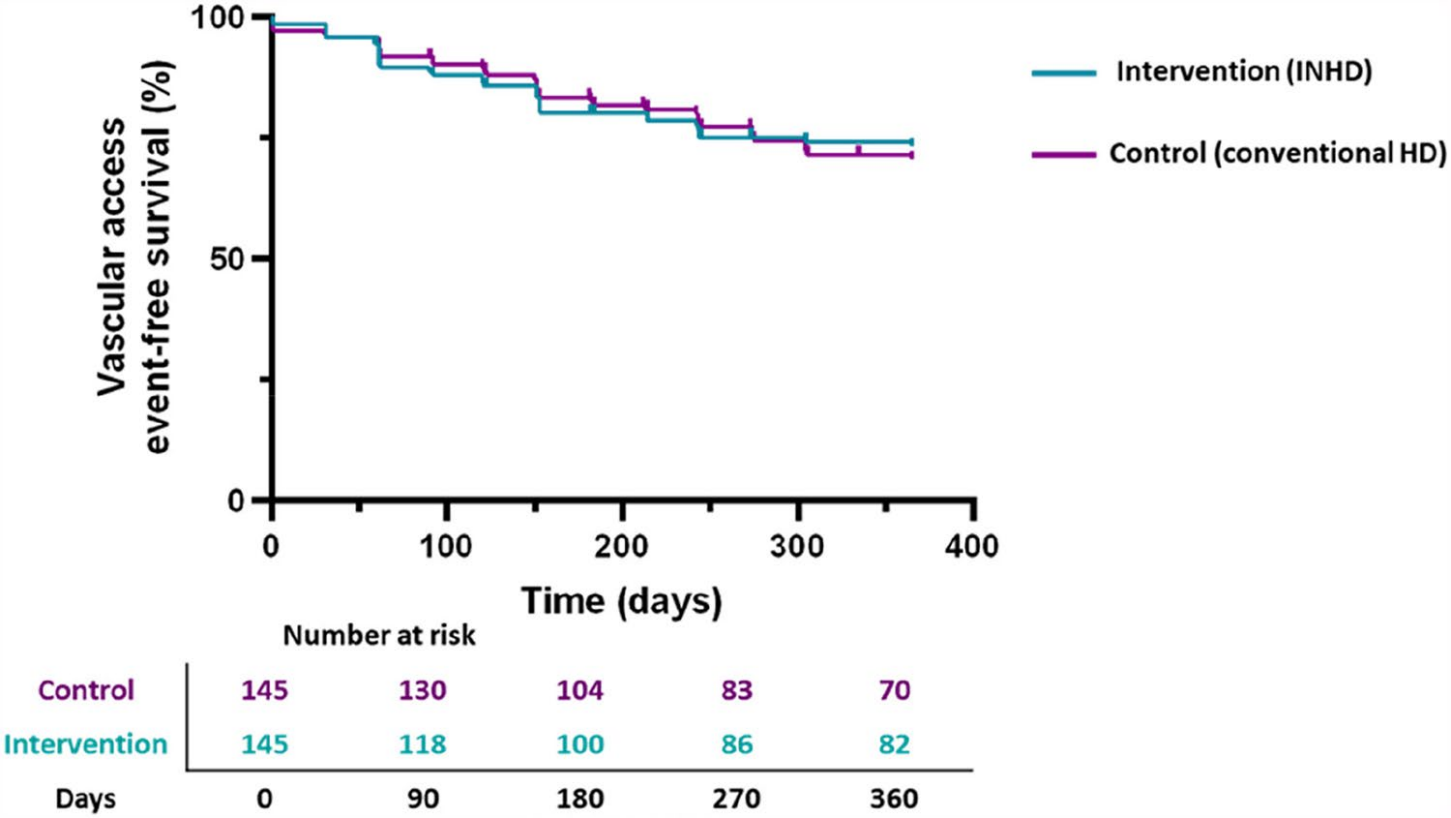
Kaplan–Meier plot demonstrating vascular access time-to-event rate, determined by reaching the composite primary outcome, for the intervention (in-centre nocturnal haemodialysis) and control (conventional daytime in-centre haemodialysis) period

The time to reaching the primary outcome was similar for both the intervention and control period (Fig. 1). The 12-month vascular access survival probability was 73.4% (95% CI 65.8–81.0%) for the intervention period and 70.6% (95% CI 62.4–78.8%) for the control period.

**Fig. 2:**
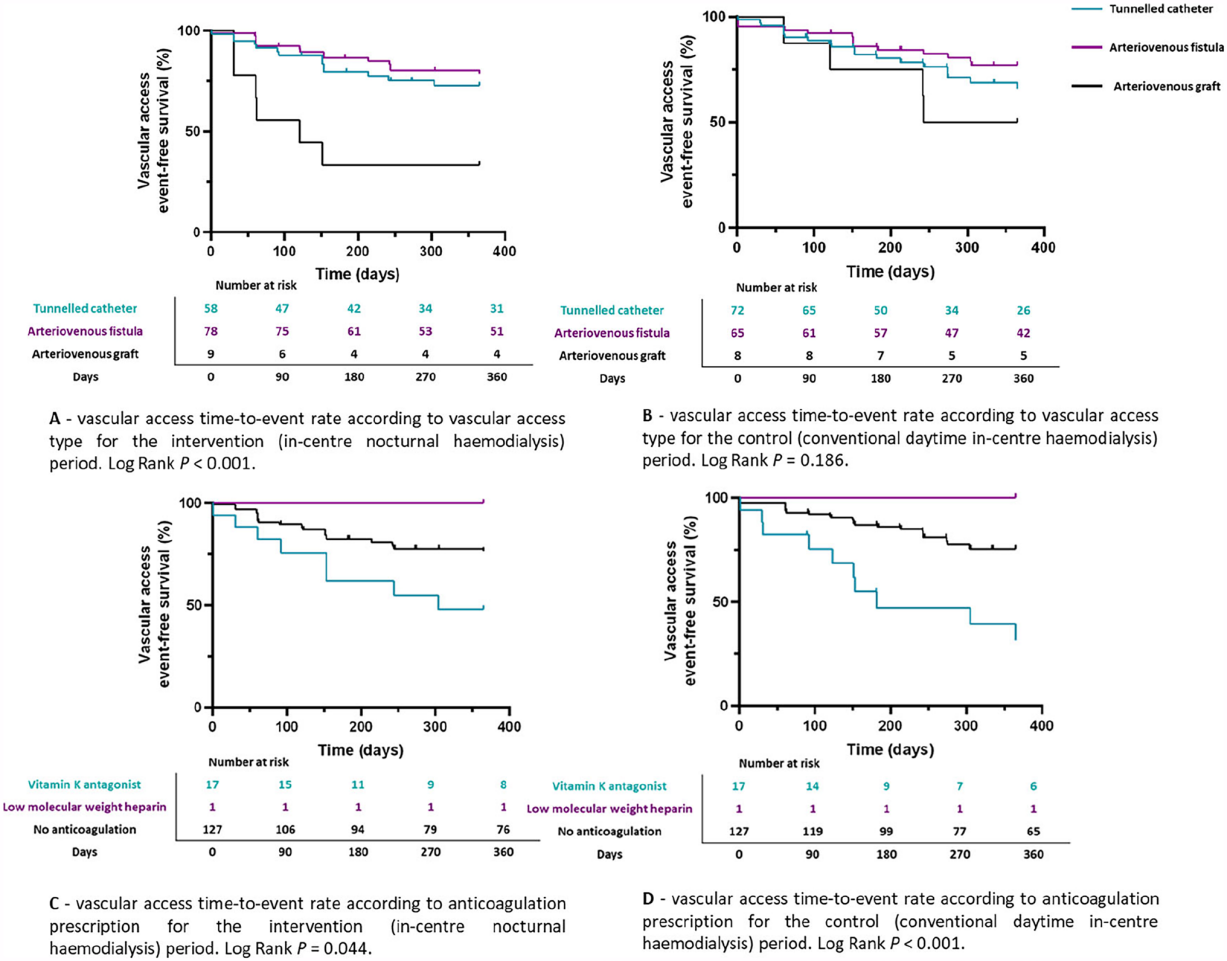
Kaplan–Meier plots demonstrating the vascular access time-to-event rate according to: the vascular access type for the intervention (**A**) and control (**B**) periods; anticoagulation prescription for the intervention (**C**) and control (**D**) periods

For the intervention period, the 12-month vascular access survival probability was similar for patients with a tunnelled catheter and AVF, whereas there was a significantly lower vascular access survival rate for patients with an AVG (*P* < 0.001, Fig. 2). There was no significant difference between the 12-month vascular access survival probability according to the type of vascular access during the control period (*P* = 0.186, Fig. 2).

For the intervention period, the 12-month vascular access survival probability was significantly lower for patients receiving regular vitamin K antagonist compared to those not receiving a regular prescription for anticoagulation (*P* = 0.044, Fig. 2). Similarly, for the control period, the 12-month vascular access survival probability was significantly lower for patients receiving regular vitamin K antagonist compared to those not receiving a regular prescription for anticoagulation (*P* < 0.001, Fig. 2).

For the control period, the 12-month vascular access survival probability was significantly lower for patients below 52 years (median) of age compared to those aged 52 years and above (*P* = 0.027). There was no significant difference between the 12-month vascular access survival probability according to age category for the intervention period (*P* = 0.350).

There were no other significant differences identified in vascular access survival probability and pre-specified patient characteristics (co-morbidities, dialysis vintage and antiplatelet prescription) within the intervention and control period.

### Exploratory secondary outcomes

The results for the secondary outcomes are reported in Table 2. A breakdown of secondary outcomes by vascular access type are reported in Supplementary Table 1.

**Table 2:** Exploratory secondary outcomes for the control and intervention period

Parameter	Control *n* = 145	Intervention *n* = 145		*P* value
*Haemodialysis safety (incidence rate)*				
Needle dislodgement	0	0		n/a
Post-haemodialysis AVF/AVG bleed	2 per 100 person-years	5 per 100 person-years		0.157
Spontaneous AVF/AVG bleed	1 per 100 person-years	2 per 100 person-years		0.564
Vascular access complication (proportion of patients)*				
Thrombosis	18 (12.4%)		28 (19.3%)	0.089
Stenosis	19 (13.1%)		27 (18.6%)	0.170
Aneurysm	4 (2.8%)		3 (2.1%)	1.000
Infection	10 (6.9%)		5 (3.4%)	0.227
*Vascular access intervention (proportion of patients*)				
Medical thrombolysis	7 (4.8%)		13 (9.0%)	0.210
Radiological intervention	28 (19.3%)		28 (19.3%)	1.000
Surgery	10 (6.9%)		9 (6.2%)	1.000
*Hospitalisation due to vascular access complication (incidence rate)*	19 per 100 person-years		18 per 100 person-years	1.000

*For vascular access complications, there were participants who had events in both the control and intervention periods: *n* = 9 for thrombosis; *n* = 10 for stenosis; *n* = 1 for aneurysm; *n* = 2 for infection

### Haemodialysis safety

There were no episodes of AVF/AVG needle dislodgement. The incidence rates of post-dialysis and spontaneous AVF/AVG bleeds were higher during the intervention than the control period, however, this did not reach statistical significance for either outcome. The post-dialysis AVF/AVG bleeds occurred in three patients during the control period and in six patients during the intervention period; one patient contributed to the incidence in both the control and intervention periods. In the intervention period, one patient had three episodes of post-dialysis AVF/AVG bleeding, and another patient in the intervention period had two episodes.

### Vascular access complication

There were more episodes of thrombosis and stenosis during the intervention than the control period, though this did not reach statistical significance for either outcome. Nine patients who experienced thrombosis during the control period also had an episode of thrombosis during the intervention period; 32.1% of the thrombosis events during the intervention were recurrences from patients in the control period (*n* = 9 of 28). Ten patients who experienced stenosis during the control period also had an episode of stenosis during the intervention period; 37.0% of the stenosis events during the intervention period were recurrences in patients in the control period (*n* = 10 of 27).

There was no significant difference in the number of patients experiencing the vascular access complication of aneurysm during the intervention and control period. There were more patients experiencing a vascular access infection during the control period (6.9%, *n* = 10) than the intervention period (3.4%, *n* = 5), however this did not reach statistical significance.

### Vascular access intervention

There were no statistically significant differences in the number of patients requiring a vascular access intervention during the intervention and control periods.

### Hospitalisation due to vascular access complication

The incidence rates of hospitalisation due to a vascular access complication were similar for both the intervention (18 per 100 person-years) and control (19 per 100 person-years) periods.

## Discussion

This retrospective cohort study demonstrates similar vascular access survival, adverse events, interventions and hospitalisations during a 12-month period of conventional daytime in-centre haemodialysis and a subsequent 12-month period of thrice-weekly extended-hours INHD. There were no deaths due to vascular access complications and no confirmed episodes of needle dislodgement. There appears to be an association between the use of an AVG and progression to a vascular access adverse event (intervention period), and an association between regular vitamin K antagonist prescription and progression to a vascular access adverse event (intervention and control periods).

This is the first study, to the authors’ knowledge, to compare vascular access survival, adverse events and safety between conventional in-centre haemodialysis and thrice-weekly extended-hours INHD. Other studies which have explored vascular access events in patients receiving extended-hours haemodialysis have involved *intensive* (i.e., > 3 times per week haemodialysis) regimens during the day or night in home and in-centre locations [4, 6]. A small, randomised crossover trial comparing conventional daytime in-centre haemodialysis to thrice-weekly, six to eight hours per session, of home haemodialysis provides a potentially comparable population [7], but there is insufficient data to explore vascular access complications [8].

There were no significant differences in the primary outcome between the control and intervention period for this retrospective cohort study. There were more episodes of post-dialysis AVF/AVG bleed, thrombosis, stenosis and medical thrombolysis in the intervention period than the control period. These secondary outcome measures did not reach statistical significance. However, there are a number of limitations (see below), and the lack of statistical significance may be a question of power. Despite not reaching statistical significance, increased episodes of such complications could be clinically significant; the nursing time and associated cost in managing these episodes, alongside the impact on the patient, have not been explored. A large proportion of the events occurred in the same patients for both the control and intervention periods. Similarly, findings from the Dialysis Outcomes and Practice Patterns Study (DOPPS) suggest that vascular access events tended to recur in the same vascular access, and that the longer a vascular access remained complication-free, the lower the risk of complications occurring [9].

A proposed mechanism for the similarity across treatment periods may relate to the frequency of the haemodialysis sessions, with patients always receiving thrice-weekly haemodialysis for both the control and intervention period. In the FHN Daily trial, participants were randomised to conventional daytime in-centre haemodialysis or to six times a week haemodialysis for 1.5–2.75 h per session. Allocation to the intervention was associated with earlier vascular access intervention compared to thrice-weekly haemodialysis (hazard ratio 1.71, 95% CI 1.08–2.73) [10]. A similar trend was observed with the FHN Nocturnal trial, with the intervention arm receiving extended-hours nocturnal HHD six times a week compared to a control arm of conventional in-centre daytime haemodialysis, but this did not reach significance (hazard ratio 1.88, 95% CI 0.97–3.64) [3]. In a case series of over 250 patients receiving extended-hours haemodialysis at home or on a nocturnal schedule in Australia, the frequency of the haemodialysis sessions was a significant predictor of vascular access event incidence, with those dialysing ≤ 3.5 times per week experiencing significantly greater vascular access survival than those dialysing > 3.5 sessions per week (log rank *P* < 0.001) [6].

It is important to acknowledge that all centres principally employed a rope ladder technique for AVF/AVG cannulation; this may have contributed to the similarity in events for the control and intervention periods. Parisotto et al. have demonstrated that the cannulation technique is associated with vascular access survival; area cannulation associates with a significantly higher risk of access failure than the rope ladder and button hole techniques [11].

There were no significant safety events such as death due to vascular access adverse events and needle dislodgement during haemodialysis for both the control and intervention periods. Needle dislodgement from AVF and AVG during haemodialysis presents a potentially life-threatening event; complete venous needle dislodgement results in blood loss at ≥ 300 mL/min (i.e., the blood flow rate) until the dialysis pump is stopped, with the potential for haemorrhagic shock and death within minutes [12]. Similar to the findings of this study, fatal needle dislodgement events are reportedly rare in the literature. Jose et al. evaluated all reported episodes of death from vascular access bleeding in Australia and New Zealand over a 14-year period; 79 people died of fatal vascular access haemorrhage between 2000 and 2013, with an incidence of 1 death for every 1250 person-years of haemodialysis [13]. Despite these low event rates, there is concern that events are underreported [14] and near-misses are not captured in routine dialysis practice [15]. As a result, it is important to acknowledge that this retrospective cohort study may not reflect *all* the safety events that have occurred at each participating site due to potential underreporting in routine clinical practice and a lack of measures to capture near-misses.

Arteriovenous grafts had a significant association with reduced vascular access survival probability during the intervention period, and vitamin K antagonist prescription had a significant association with reduced vascular access survival during the intervention and the control periods. These findings need to be interpreted with caution due to the low numbers within the study population. However, similar observations have been reported in the literature. Arteriovenous grafts have poorer long-term patency [16] and an increased risk of venous thromboembolism [17] compared to AVF. In a retrospective cohort study of individuals receiving nocturnal home haemodialysis, the use of an AVG was a significant predictor of the occurrence of vascular access adverse events and was associated with an increased frequency of events [18]. The UK Kidney Association guidelines recommend favouring AVF formation over AVG whenever possible due to the increased complication rate and poorer outcomes associated with AVGs [19]. With regard to regular vitamin K antagonist prescription, observational studies have demonstrated that warfarin use is associated with poorer primary graft patency [20] and anticoagulation use is associated with higher wound infection rates for a study population utilising both AVF and AVGs [21]. In a placebo-controlled RCT, warfarin use did not significantly impact graft survival and resulted in significantly more major bleeding events [22].

There was also a significant association between age and the 12-month vascular access survival probability during the control period, whereby younger patients (< 52 years) had a lower vascular access survival probability than those ≥ 52 years. This association did not persist for the intervention period. These findings are not reflected in the literature, whereby increasing age has been identified as a risk factor for vascular access dysfunction [23–25]. It also seems implausible that the impact of age would reduce with time as the absence of a significant association in the intervention period suggests. Thus, the significant association between vascular access survival probability and age observed in the control period may reflect the impact of an unaccounted for confounding factor(s), alongside the limitations of the dataset and study design.

There are a number of limitations that must be considered. The findings are limited to adult prevalent haemodialysis populations. The retrospective design introduces bias, including: potential confounding factors, e.g., patients with well-established vascular access may be more likely to be offered INHD in routine clinical practice; information bias due to the collection of data from clinical records; selection bias favouring INHD, with the study cohort being substantially younger (median age of 52 years) than the typical age for people starting dialysis in the UK (median age of 63 years) [26]. However, it is not uncommon for INHD cohorts to be younger, with average ages reported at less than 60 years old [27, 28]. All the demographic data were collected when the patient commenced INHD, and changes in routine patient care are unknown. Vascular access vintage was not an included covariate. Data collection from retrospective review of medical records was limited by the clinical information available, e.g., causes of death outside of the primary outcome, and individual cannulation techniques were not retrievable.

The study design, whereby all participants received conventional daytime in-centre haemodialysis followed by INHD, introduces bias, including: *period effect*, e.g., there may be an overall decline in a patient’s condition and vascular ageing with time; *carryover effect*, whereby the impact of receiving one type of dialysis has lasting influence on the occurrence of events in the intervention period; and *sequence effect*, whereby there may be a specific interaction of receiving conventional in-centre daytime dialysis prior to INHD [29]. There was an increased number of events for post-dialysis AVF/AVG bleed, thrombosis and stenosis during the intervention (INHD) period, with a substantial contribution from the same patients in both follow-up periods. This may suggest an impact of both a period and a carryover effect. The observational design of the study also introduces *immortal time bias* as all the participants were required to survive a period of time on haemodialysis without experiencing an event before being eligible for the data collection control and intervention periods.

Nonetheless, the results fill an important gap in understanding vascular access complications in an extended-hours, thrice-weekly INHD cohort. Data collection involved three dialysis centres across two continents, increasing the generalisability of the findings. Although the findings are not definitive, they demonstrate that concerns regarding vascular access survival and safety events should not inhibit dialysis centres from offering an INHD programme. The findings could also be used as a preliminary dataset regarding INHD safety when counselling patients about dialysis choices. Further work is needed, primarily the collection of prospective data of vascular access survival, adverse events and safety, comparing INHD to daytime in-centre haemodialysis. The evaluation of safety needs to capture near-misses alongside confirmed events.

## Conclusion

There does not appear to be an increased risk to vascular access survival, adverse events, and safety for INHD compared to daytime in-centre haemodialysis. The complexity of an individual’s past medical history, particularly their type of vascular access and requirement for regular anticoagulation, is associated with a reduced vascular access survival probability, rather than the length and timing of the in-centre dialysis session.

## Supplementary Information

Below is the link to the electronic supplementary material.

Supplementary file1 (DOCX 70 KB)

Supplementary file2 (DOCX 19 KB)

## Data Availability

Deidentified individual participant data collected for the study, and a data dictionary defining each field in the set, will be made available to others on specific request to the chief investigator (JOB) and corresponding author (KLH) provided all regulatory and data sharing approvals are obtained.
