# Supplementary figures and images for: Exploring vascular access survival in prevalent thrice-weekly in-centre nocturnal haemodialysis patients
Source: J Nephrol. 2025 Sep 26;38(9):2651–61. doi: 10.1007/s40620-025-02431-1 (PMC12711934; doi:10.1007/s40620-025-02431-1)

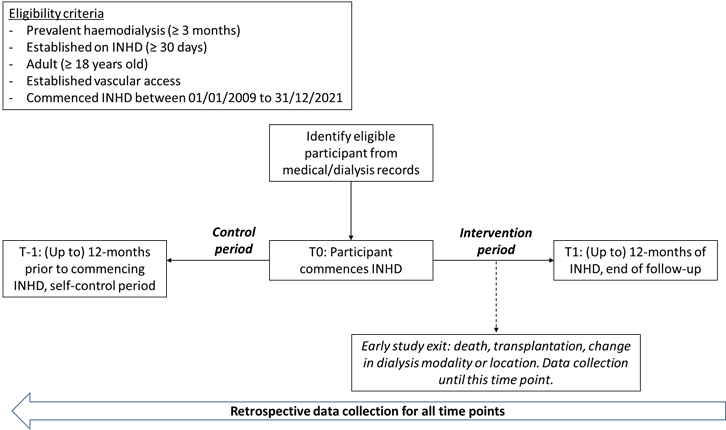


Supplementary Figure - Overview of study design and eligibility criteria

Supplement: Supplementary file 1 — Supplementary file1 (DOCX 70 KB) [file 40620_2025_2431_MOESM1_ESM.docx]
