# Supplementary material for: Exploring vascular access survival in prevalent thrice-weekly in-centre nocturnal haemodialysis patients
Source: J Nephrol. 2025 Sep 26;38(9):2651–61. doi: 10.1007/s40620-025-02431-1 (PMC12711934; doi:10.1007/s40620-025-02431-1)
Supplement: Supplementary file 2 — Supplementary file2 (DOCX 19 KB) [file 40620_2025_2431_MOESM2_ESM.docx]

Supplementary Table 1 – Secondary outcomes according to haemodialysis access type for the control and intervention period

| **Parameter** | **Control n = 145** | **Intervention n = 145** |
| --- | --- | --- |
| ***Haemodialysis safety (incidence rate)***  Needle dislodgement  Post-haemodialysis AVF/AVG bleed   - AVF - AVG   Spontaneous AVF/AVG bleed   - AVF - AVG | 0  3 per 100 person-years  0  2 per 100 person-years  0 | 0  7 per 100 person-years  11 per 100 person-years  3 per 100 person-years  0 |
| ***Vascular access complication (proportion of patients)***  Thrombosis   - AVF - AVG - Tunnelled catheter   Stenosis   - AVF - AVG - Tunnelled catheter   Aneurysm   - AVF - AVG - Tunnelled catheter   Infection   - AVF - AVG - Tunnelled catheter | 7/65 (10.8%)  0/8  11/72 (15.3%)  8/65 (12.3%)  3/8 (37.5%)  8/72 (11.1%)  3/65 (4.6%)  1/8 (12.5%)  0/72  0/65  0/65  10/72 (13.9%) | 8/78 (10.3%)  4/9 (44.4%)  16/58 (27.6%)  12/78 (15.4%)  5/9 (55.6%)  10/58 (17.2%)  3/78 (3.8%)  0/9  0/58  2/78 (2.6%)  0/9  3/72 (4.2%) |
| ***Vascular access intervention (proportion of patients***)  Medical thrombolysis   - AVF - AVG - Tunnelled catheter   Radiological intervention   - AVF - AVG - Tunnelled catheter   Surgery   - AVF - AVG - Tunnelled catheter | 0/65  0/8  7/72 (9.7%)  11/65 (16.9%)  2/8 (25%)  15/72 (20.8%)  5/65 (7.7%)  1/8 (12.5%)  4/72 (5.6%) | 2/78 (2.6%)  1/9 (11.1%)  10/58 (17.2%)  12/78 (15.4%)  5/9 (55.6%)  11/58 (19.0%)  2/78 (2.6%)  4/9 (44.4%)  3/58 (5.2%) |
| ***Hospitalisation due to vascular access complication (incidence rate)***   - AVF - AVG - Tunnelled catheter | 12 per 100 person-years  0  30 per 100 person-years | 16 per 100 person-years  56 per 100 person-years  15 per 100 person-years |
